# Ductal carcinoma in situ and sentinel lymph node metastasis in breast cancer

**DOI:** 10.1186/1477-7819-8-6

**Published:** 2010-01-27

**Authors:** Keiichiro Tada, Akiko Ogiya, Kiyomi Kimura, Hidetomo Morizono, Kotaro Iijima, Yumi Miyagi, Seiichiro Nishimura, Masujiro Makita, Rie Horii, Futoshi Akiyama, Takuji Iwase

**Affiliations:** 1 Department of Breast Surgery, Cancer Institute Hospital, Tokyo, Japan; 2 Department of Pathology, The Cancer Institute of the Japanese Foundation for Cancer Research, Tokyo, Japan

## Abstract

**Background:**

The impact of sentinel lymph node biopsy on breast cancer mimicking ductal carcinoma in situ (DCIS) is a matter of debate.

**Methods:**

We studied the rate of occurrence of sentinel lymph node metastasis in 255 breast cancer patients with pure DCIS showing no invasive components on routine pathological examination. We compared this to the rate of occurrence in 177 patients with predominant intraductal-component (IDC) breast cancers containing invasive foci equal to or less than 0.5 cm in size.

**Results:**

Most of the clinical and pathological baseline characteristics were the same between the two groups. However, peritumoral lymphatic permeation occurred less often in the pure DCIS group than in the IDC-predominant invasive-lesion group (1.2% vs. 6.8%, p = 0.002). One patient (0.39%) with pure DCIS had two sentinel lymph nodes positive for metastasis. This rate was significantly lower than that in patients with IDC-predominant invasive lesions (6.2%; p < 0.001).

**Conclusions:**

Because the rate of sentinel lymph node metastasis in pure DCIS is very low, sentinel lymph node biopsy can safely be omitted.

## Introduction

The technique of sentinel lymph node biopsy is used worldwide as a surgical treatment for breast cancer [1,2]. This procedure can accurately determine lymph node metastasis [3,4]. Therefore morbid axillary dissection can be safely avoided when sentinel lymph nodes are free from cancer [5,6].

The primary indication for sentinel lymph node biopsy is invasive breast cancer, which has the potential of metastasizing to the regional lymph nodes. On the other hand, ductal carcinoma in situ (DCIS), which has no invasive foci and is isolated from the interstitium, is not believed to metastasize to the lymph nodes [7].

The determination of DCIS requires thorough examination of surgical materials, and very infrequent lymph node metastases are observed in cases of DCIS that show no invasive components on routine pathological examination [8]. Furthermore, thorough examination of the sentinel lymph nodes, which are the most likely candidates for metastasis, is feasible. In these situations, some investigators have argued that more than a few cases of pure DCIS are accompanied by sentinel lymph node metastasis, and the indications for sentinel lymph node biopsy should be extended not only to cases with invasive cancer, but also to those with pure DCIS [9]. However, others have argued that the incidence of lymph node metastasis in pure DCIS is still very low, and sentinel lymph node biopsy can be safely avoided in these cases [10,11].

In this article, we studied the incidence of sentinel lymph node metastasis in cases of pure DCIS. Furthermore, we compared this incidence with that of predominant intraductal-component (IDC) breast cancer with invasive foci equal to or less than 0.5 cm in size. Then we addressed the question of whether sentinel lymph node biopsy is required in cases of pure DCIS.

## Materials and methods

### Patients and study design

We searched our surgical records from December 2006 to June 2008 for patients with a histology of pure DCIS for our study. Pure DCIS was determined histopathologically as intraductal carcinoma without stromal invasion. Inclusion criteria were as follows: curative surgical treatment, performance of sentinel lymph node biopsy, and no primary chemotherapy. Patients with metachronous ipsilateral breast cancer were excluded. Furthermore, we also searched for patients having an IDC-predominant invasive lesion with the same profile as mentioned above. IDC-predominant invasive lesions are those with a predominant IDC including one or more invasive foci, each of which is not more than 0.5 cm in size.

### Sentinel lymph node biopsy procedures

The method for sentinel lymph node biopsy using a radioactive agent has been described elsewhere [12]. Briefly, the radioactive tracer used was 1.5 mCi/ml of 99mTc-phytate (Daiichi Radioisotope Laboratories, Ltd). The radioactive tracer was injected into the intradermal space in the area of the tumor and the retro-tumoral space. The tracer was injected the day prior to surgery. In all cases, a lymphoscintigraphy was obtained one hour after injection. Additionally, vital dye (indigocarmine) was injected intradermally in the peri-tumoral space just before surgery.

### Histopathological procedures

Surgical materials from breast-conserving surgery were sectioned at 0.5 cm intervals, and each section was examined histologically. Surgical materials from mastectomy were cut at several representative sections in order to study the histopathological characteristics.

Sentinel lymph nodes were sectioned at 0.2 cm intervals, and examinations were based on frozen sections in most cases. Whether or not metastasis was present was determined intraoperatively. Immunohistochemistry was not used for analysis.

### Statistical analysis

Frequency analysis was performed with Fisher's exact test. The difference in continuous variables was evaluated using Student's t-test. A significance level of 0.05 was used for statistical tests, and two-tailed tests were applied. Calculations were performed using SPSS 16.0J for MAC (SPSS Japan Inc. Tokyo).

## Results

### Study population

From December 2006 to June 2008, 1919 surgical and pathological records were registered. Among these, 1302 cases had sentinel lymph node biopsy and no primary chemotherapy. In this cohort, 255 patients had pure DCIS and 177 patients had an IDC-predominant invasive lesion. During the same period, there were 42 cases who had pure DCIS without sentinel lymph node biopsy.

### Patient characteristics

The patients' characteristics are summarized in Table 1.

**Table 1:** Patient characteristics

		Pure DCIS	IDC predominant invasive lesion	P-value
Mean Age	(Range)	51.2(29-81)	51.9(27-86)	*NS

Lymphatic permeation		3(1.2%)	12(6.8%)	0.002

Breast-conserving surgery		147(57.6%)	96(54.2%)	NS

ER	positive	194(74.9%)	133(76.8%)	NS
	negative	49(19.2%)	36(20.3%)	
	unknown	15(5.9%)	5(2.8%)	

PgR	positive	162(62.8%)	114(65.5%)	NS
	negative	81(31.4%)	55(31.6%)	
	unknown	15(5.8%)	5(2.8%)	

Median number of removed nodes		2	2	NS

* NS; Not Specific

Most clinical and pathological baseline characteristics showed no differences between the groups, including age, estrogen receptor status, progesterone receptor status, removed sentinel nodes, and surgical procedures. However, the frequency of peritumoral lymphatic invasion was higher in the IDC-predominant invasive-lesion group than in the pure DCIS group (6.8% vs 1.2%: p = 0.002).

### Patients with a positive sentinel node biopsy

One patient (0.39%) with pure DCIS had two sentinel lymph nodes positive for metastasis, whereas 6.2% of the patients with IDC-predominant invasive breast cancer had positive sentinel lymph nodes. Therefore, the risk of lymph node metastasis was significantly lower in the pure DCIS group than in the IDC-predominant invasive-lesion group, with a statistical significance of p < 0.001. The contingency table for the two groups is shown in Table 2.

**Table 2:** Contingency table

	Node-positive	Node-negative	Total
Pure DCIS	1	254	255
IDC predominant invasive lesion	11	166	177

	12	420	432

p < 0.001

The major characteristics of node-positive patients with pure DCIS or IDC-predominant invasive lesions are summarized in Table 3. The patient with pure DCIS had exclusive breast-conserving surgery, with a slight positive surgical margin, and received radiation therapy. Among the 11 patients who had an IDC-predominant invasive lesion with positive sentinel nodes, 4 patients had dislocation of cancer cells along the biopsy scar.

**Table 3:** Patients with positive nodes

	Age	Clinical presentation	Histology	Comedonecrosis	Number of positive nodes	Size of metastasis in nodes	Lymphatic permeation	Tumor dislocation
1	46	US-detected mass	Pure DCIS	no	2	macro	None	none
2	48	Palpable mass	IDC predominant invasive lesion	no	1	micro	Present	yes
3	29	Palpable mass	IDC predominant invasive lesion	yes	1	micro	Present	yes
4	48	Nipple discharge	IDC predominant invasive lesion	no	1	micro	None	yes
5	54	Calcification on MMG	IDC predominant invasive lesion	no	1	ND	None	none
6	45	Palpable mass	IDC predominant invasive lesion	yes	2	macro	None	none
7	45	Calcification on MMG	IDC predominant invasive lesion	yes	1	micro	None	none
8	53	Palpable mass	IDC predominant invasive lesion	no	2	micro	None	none
9	54	Nipple discharge	IDC predominant invasive lesion	yes	1	macro	None	none
10	65	Palpable mass	IDC predominant invasive lesion	yes	1	macro	None	none
11	50	Palpable mass	IDC predominant invasive lesion	yes	1	macro	None	none
12	44	Palpable mass	IDC predominant invasive lesion	no	1	micro	None	yes

Abbreviations: US; Ultrasonography, MMG; Mammography, ND; Not Determined

## Discussion

In this study, we found that the incidence of sentinel lymph node metastasis in cases of pure DCIS was 0.39%. This incidence was significantly lower than that in cases of IDC-predominant invasive tumors (0.39% vs. 6.2%; p < 0.001). Therefore, our data suggest that sentinel lymph node biopsy can be avoided in cases of pure DCIS.

Many publications concerning this issue have reported only the rate of sentinel lymph node metastasis in pure DCIS. We also calculated the rate of metastasis in IDC-predominant invasive lesions. We believe that the relevance of the metastasis rate in pure DCIS is supported by comparing data concerning IDC-predominant invasive lesions. Furthermore, we can estimate the rate of sentinel lymph node metastasis in lesions mimicking DCIS clinically.

The issue of pure DCIS and sentinel node biopsy is associated with two major problems: one is that preoperative diagnosis of pure DCIS is difficult, and the other is that postoperative definitive diagnosis of pure DCIS is also difficult.

It is well known that preoperative diagnoses of DCIS based on core needle biopsy are likely to be underestimated. Rates of diagnosis range from 8.3% to 43.6% [8,13,14]. Preoperative core needle biopsy does not guarantee that the entire lesion is without stromal invasion. Furthermore, having less than 0.5 cm of stromal invasion increases the incidence of sentinel lymph node metastases [15,16]. As a result, many investigators insist that sentinel lymph node biopsy should be encouraged when DCIS-like tumors are large enough to be palpable or when tumors require total mastectomy.

Furthermore, the postoperative pathological diagnosis of pure DCIS does not always guarantee the absence of lymph node metastasis. For many years, it has been believed that DCIS is associated with the absence of lymph node metastasis, that axillary dissection in DCIS could be omitted, and that cases of lymph node metastasis in DCIS are associated with invasive lesions that are too small to be detected by the usual pathological examination. However, in regular clinical practice the detection of minimal stromal invasion is quite difficult. Although sentinel lymph node biopsy is effective in DCIS, we suggest that the application of sentinel node biopsy to all DCIS cases should be avoided. That is because, although sentinel node biopsy is less morbid than axillary dissection, the procedure is not completely free from morbidity [17].

We believe that Moore et al., who encouraged the use of sentinel lymph node biopsy in pure DCIS, does not argue that sentinel lymph node biopsy should be carried out in all cases of pure DCIS [9]. In their literature, only 22% of all DCIS cases had sentinel lymph node biopsy. The relatively high rate of axillary lymph node metastases in their study can be associated with this selection.

In our series there was one case of pure DCIS with positive sentinel nodes. This case underwent a partial mastectomy, and the surgical margin was slightly positive. Preoperative mammography, ultrasonography, and MRI did not reveal any other abnormal lesions besides the main tumor. However two sentinel nodes were positive for cancer and both metastases were larger than 2 mm. We think that this was an extremely rare case. Although some authors encourage the preservation of axillary nodes in cases of pure DCIS with positive sentinel nodes [10], an axillary dissection was performed in this patient.

There is much debate concerning the association between preoperative invasive procedures for diagnosis and the likelihood of lymph node metastases. Displacement of cancer cells around the main tumor is common, and frequencies from 28% to 32% have been reported previously [18,19]. Moreover, there is the possibility that displacement can cause the migration of cancer cells to lymph nodes[20]. However, the prognostic significance of this migration is uncertain. Previous studies show that large gauge needle biopsy does not affect the survival risk [21,22]. Much more discussion and careful studies on this issue are necessary.

Our study has a considerable limitation. Our series could miss cases of micrometastases or isolated tumor cells (ICT) in sentinel nodes. In order to avoid this problem, the sentinel nodes should be sectioned at intervals of at least 0.15 mm and immunohistochemistry should be applied to sections at different levels. These analyses should be performed on permanent paraffin sections. Although the clinical significance of micrometastases and ICT in DCIS has been unclear [23], the latest report has shown that micrometastases or ICT may decrease the probability of survival in invasive breast cancers [24].

In conclusion, we found that the incidence of sentinel lymph node metastasis in cases of pure DCIS was 0.39%. This incidence was lower than that in IDC-predominant invasive lesions. Therefore, we believe that sentinel lymph node biopsy in pure DCIS can be safely omitted.

## Competing interests

The authors declare that they have no competing interests.

## Authors' contributions

KT designed the study, researched the literature, and drafted the manuscript. FA, RH, and AO contributed to the histopathological analyses. KK, HM, KI, YM, SN, MM, and TI participated in the study design and coordination, and helped to collect data.
